# Improvement of Inflammation through Antioxidant Pathway of Gardeniae Fructus 50% EtOH Extract (GE) from Acute Reflux Esophagitis Rats

**DOI:** 10.1155/2020/4826176

**Published:** 2020-02-24

**Authors:** Soo Hyun Kim, Mi-Rae Shin, Ah Reum Lee, Bu-Il Seo, Hae-Jin Park, Seong-Soo Roh

**Affiliations:** ^1^Department of Herbology, College of Korean Medicine, Daegu Haany University, 136, Shinchendong-ro, Suseong-gu, Deagu 42158, Republic of Korea; ^2^Department of Beauty Design, Hosan University, Gyeongsan, Republic of Korea; ^3^Faculty of Herbal Cuisine and Nutrition, Daegu Haany University, Gyeongsan, Republic of Korea

## Abstract

Gardeniae Fructus 50% EtOH extract (GE) is a traditional herb that has been used to treat a variety of diseases. In this study, we investigate the antioxidant, anti-inflammatory, and antiapoptotic properties of GE on acute reflux-induced esophagitis (RE) model in rats. 2,2′-Azino-bis (3-ethylbenzothiazolin-6-sulfonic acid) (ABTS) and 2,2-diphenyl-1-picrylhydrazyl (DPPH) radical scavenging assays were performed to determine the antioxidant activity of GE. GE was given orally at 50 and 100 mg/kg body weight 1h 30 min prior to RE induction. And its effect was assessed in comparison with RE control and normal groups. The administration of the extract of the GE showed remarkable protection of mucosal damage in esophageal tissue, and the histologic observation showed that the gastric lesion was improved. Increased reactive oxygen species (ROS) levels in the serum were diminished by GE treatment. The antioxidative biomarkers including nuclear factor-erythroid 2-related factor 2 (Nrf-2), heme oxygenase-1 (HO-1), superoxide dismutase (SOD), catalase, and glutathione peroxidase (GPX) were significantly increased. GE administration significantly reduced the inflammatory protein expression through MAPK-related signaling pathways and the nuclear factor-kappa B (NF-*κ*B) pathway. These results suggest that GE protects the esophagus mucosal membrane by attenuating oxidative stress and inflammatory response under reflux esophagitis condition through the antioxidant pathway. Therefore, it is suggested that GE may be a potential remedy for the treatment of reflux esophagitis.

## 1. Introduction

Reflux esophagitis (RE) is an inflammation of the esophagus caused by reflux of gastric contents due to damage of the lower esophagus. It is characterized by a burning pain in the chest (so-called heartburn) and nausea after meals, and it includes reflux disease [1, 2]. Though the exact mechanism underlying the occurrence of reflux esophagitis remains unknown, alcohol, smoking, obesity, stress, and various etiologies have been identified as the source of disease pathogenesis [3]. Reflux of gastric contents causes inflammation and ulceration, leading to the destruction of normal esophageal tissue [4, 5]. According to the results of a recent study, oxidative stress has been shown to be a crucial pathogenesis of reflux esophagitis [6, 7]. At the cellular level, reflux esophagitis causes acidosis and necrosis of the esophageal tissue proliferation of hydrogen ions to the mucosa due to oxidative stress [8]. All of these activities are activated by inflammatory and neutrophils decrease and reactive oxygen species (ROS), resulting in the penetration of active oxygen into all cells and the release of cytokines. Because the overproduction of ROS causes inflammation, antioxidants have been shown to block free radicals and prevent esophageal mucosal damage [9]. Due to oxidative stress, endothelial cells produce a large number of ROS through nicotinamide adenine dinucleotide phosphate (NADPH) in cell membranes. Intrinsic apoptosis induced by the explosion of ROS and inflammatory cytokines can cause mitochondrial energy metabolism disorders and lead to cell damage [10]. Currently, the existing treatments for RE include medical therapy like antacids, H2-receptor antagonists, proton pump inhibitors (PPI), and surgical therapy [11]. The Gardeniae Fructus has been used in traditional Chinese medicine for various treatments such as cholagogue, anti-inflammatory, and antipyretic effects [12].

Gardeniae Fructus has been widely used as an herbal medicine for inflammation-related diseases and it has shown various pharmacological abilities such as anti-inflammatory effects and reduction of oxidative stress [13, 14]. There has been no report on improvement through Gardeniae Fructus 50% EtOH extract (GE) treatment in reflux-induced esophagitis. Therefore, we evaluated the antioxidative effects of the Gardeniae Fructus and investigated the effects on the reflux-induced esophagitis rats to explore the improvement effects of oxidative stress-related inflammation.

## 2. Materials and Methods

### 2.1. Materials

The protease inhibitor mixture solution and ethylenediaminetetraacetic acid (EDTA) were purchased from Wako Pure Chemical Industries, Ltd. (Osaka, Japan). Phenylmethylsulfonyl fluoride (PMSF) was purchased from Sigma Chemical Co. (St. Louis, MO, USA). 2′,7′-Dichlorofluorescein diacetate (DCF-DA) was obtained from Molecular Probes (Eugene, OR, USA). The pierce bicinchoninic acid (BCA) protein assay kit was obtained from Thermo Scientific (Rockford, IL, USA). ECL Western Blotting Detection Reagents and pure nitrocellulose membranes were supplied by GE Healthcare (Piscataway, NJ, USA). Rabbit polyclonal antibodies against nuclear factor-kappa B p65 (NF-*κ*Bp65; 1 : 1,000, SC-372), nuclear factor-erythroid 2-related factor 2 (Nrf-2; 1 : 1,000, SC-7228), heme oxygenase-1 (HO-1; 1 : 1,000, SC-10789), superoxide dismutase (SOD; 1 : 1,000, SC-11407), catalase (1 : 1,000, SC-50508), glutathione peroxidase-1/2 (GPx-1/2; 1 : 1,000, SC-30147), p47^phox^ (1 : 1,000, SC-14015), Rac-1 (1 : 1,000, SC-217), Bax (1 : 1,000, SC-7480), and Bcl-2 (1 : 1,000, SC-7382); goat polyclonal antibodies against tumor necrosis factor-alpha (TNF-*α*; 1 : 1,000, SC-1351), interleukin-1beta (IL-1*β*; 1 : 1,000, SC-1252); mouse monoclonal antibodies against cyclooxygenase-2 (COX-2; 1 : 1,000, SC-19999), inducible nitric oxide synthase (iNOS, 1 : 1,000, SC-7271), phosphor-inhibitory kappa *b* alpha (*p*-I*κ*b*α*; 1 : 1,000, SC-8404), phosphor-extracellular signal-regulated kinase 1/2 (p-ERK1/2; 1 : 1,000, SC-7383), phosphor-p38 (p-p38; 1 : 1,000, SC-7973), phosphor c-Jun N-terminal kinase (p-JNK, 1 : 1000, SC-6254), cytochrome c (1 : 1,000, SC-13156), histone (1 : 1,000, SC-8030), and *β*-actin (1 : 1,000, SC-4778) were purchased from Santa Cruz Biotechnology, Inc. (Santa Cruz, CA, USA). Monoclonal antibody against c-Jun (1 : 1000, #2315) and polyclonal antibody against c-Fos (1 : 1,000, #4384) were obtained from Cell Signaling Technology Inc. (Cell Signaling, MA, USA). Mouse monoclonal antibodies against caspase-3 (1 : 1,000,3004-100) were purchased from BioVision Inc. (Mountain View, CA, USA). Rabbit polyclonal anti-reduced nicotinamide adenine dinucleotide phosphate oxidase 4 (NOX4) was purchased from Life SpanBioSciences (Seattle, WA, USA). Mouse monoclonal antibodies against survivin (1 : 1,000, NB 500-205) were purchased from Novus Biologicals (Littleton, CO, USA). Rabbit anti-goat (1 : 3,000, SC-2774), goat anti-rabbit (1 : 3,000, SC-2004), and goat anti-mouse (1 : 3,000, SC-2005) immunoglobulin G (IgG) horseradish peroxidase- (HRP-) conjugated secondary antibodies were acquired from Santa Cruz Biotechnology, Inc. (Santa Cruz, CA, USA). All other used chemicals and reagents were of an analytical grade and commercially available (Sigma Aldrich Co., Ltd., USA).

### 2.2. Test Material Preparation

Gardeniae Fructus was purchased from Ominherb Co. (Yeongcheon, Korea). The dried slices of Gardeniae Fructus (30 g) were extracted with 50% EtOH (300 mL) at room temperature for 24h and the solvent was evaporated *in vacuo* to gain powder with a yield of 23.12%, by weight.

### 2.3. Analysis of Gardeniae Fructus by HPLC Chromatogram

**Figure 1:**
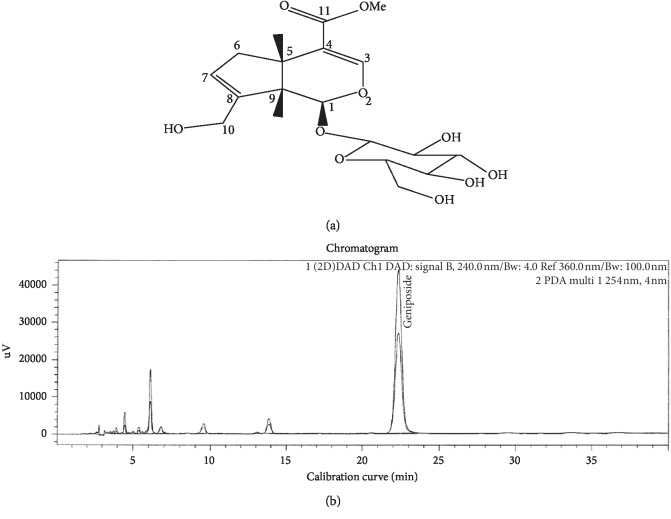
HPLC profile of GE. Geniposide (C17H24O10:388.37) (a), HPLC profile of geniposide (b).

The 50% EtOH extract of Gardeniae Fructus (5.11 mg) was dissolved in 50 mL of 70% ethanol with multivortexing. We injected 1 *μ*L of the sample into high-performance liquid chromatography (HPLC) using Agilent analytical 0.25 × 460 mm, 5 microns, with a column temperature of 30°C, mobile phase component acetonitrile—water (v/v, 9 : 91). The flow rate was 1.0 mL/min. The UV absorbance from 240 nm was monitored using an Agilent 2960 series photodiode array detector from Waters Co. (Manchester, UK). All peaks were assigned by carrying out coinjection tests with authentic samples and comparing those with the UV spectral data. The major component of Gardeniae Fructus was geniposide, and the measurement was repeated two times. Representative HPLC results are illustrated in Figure 1.

### 2.4. DPPH Radical Scavenging Activity of GE

The antioxidant activity determination of GE was performed by the DPPH radical scavenging according to the method of Hatano et al. [15]. The reduction of the stable purple free radical DPPH to the yellow hydrazine is achieved by trapping the unpaired electrons, and the degree of discoloration indicates the scavenging activity of samples [16]. 100 *μ*L of an ethanolic solution of GE (blank: 100 *μ*L of ethanol) was added to 100 *μ*L of an ethanolic solution of DPPH (60 *μ*M) using a 96-well microtiter plate. The ascorbic acid (standard sample) and GE were prepared for eight concentrations (0.5, 1, 2.5, 5, 10, 25, 50, 100, 250, 500, and 1000 *μ*g/mL). After mixing gently and leaving the mixture to stand for 30 min at room temperature, the optical density was determined using a Microplate Reader, model infinite M200 PRO (Tecan, Austria). The mixture was measured spectrophotometrically at 540 nm. The antioxidant activity of 4 evidence-based complementary and alternative medicine samples was expressed in terms of the IC_50_ (micromolar concentration required to inhibit DPPH radical formation by 50%, calculated from the log-dose inhibition curve).

(1) $$\begin{array}{c} \text{DPPH radical scavenging activity}\left(\%\right)=\left[1-\left(\frac{\text{Asample}}{\text{Ablank}}\right)\right]\cdot 100. \end{array}$$

### 2.5. ABTS Radical Scavenging Activity of GE

The ABTS radical scavenging activity of the different extracts was measured according to the modified method of Re et al. [17]. The ABTS stock solution was dissolved in water to a 7.4 mM concentration. The ABTS radical cation was produced by reacting ABTS stock solution with 2.45 mM potassium persulfate and allowing the mixture to stand for 14h at room temperature in the dark. The ABTS solution was diluted with ethanol to obtain an absorbance of 0.70 ± 0.02 at 415 nm. After adding 95 *μ*L of diluted ABTS solution (*A*415 nm = 0.70 ± 0.02) to 5 *μ*L of sample, we left the mixture at room temperature for 15 min in the dark. The absorbance at 415 nm was measured using a Microplate Reader, model infinite M200 PRO (Tecan, Austria). The blank was prepared in the same manner, except that distilled water was used instead of the sample.

(2) $$\begin{array}{c} \text{ABTS radical scavenging activity}\left(\%\right)=\left[1-\left(\frac{\text{Asample}}{\text{Ablank}}\right)\right]\cdot 100. \end{array}$$

### 2.6. Experimental Animals and Treatment

Animal experiments were carried out according to the “Guidelines for Animal Experimentation” approved by the Ethics Committee of the Daegu Haany University (Approval number 2018-003). Six-week-old male Sprague-Dawley rats were purchased from Samtako (Eumseong, Korea). Rats were maintained under a 12 h light/dark cycle and housed at a controlled temperature (24 ± 2°C) and humidity (about 55%). After adaptation (1 week), a total of 24 SD rats were randomly divided into 4 groups (*n* = 6 per group). The rats were fasted for 18h prior to surgical procedures and kept in raised mesh-bottom cages to prevent coprophagy but were provided free access to food thereafter. The rats were anaesthetized with an injection of Zoletil 0.75 mg/kg (Virbac S. A., France). A midline laparotomy was performed to expose the stomach; both the pylorus and the transitional junction between the forestomach and the corpus were exposed and then ligated with a 2-0 silk thread without a pyloric ring, employing the method originally proposed by Omura et al. [18]. Group one included normal rats (Nor). Group two included RE control rats (Con). Group three included the RE control rats treated with GE 50 mg/kg (GL). Group four included the RE control rats treated with GE 100 mg/kg (GH). The normal and RE control rat groups were given water, while the other groups were orally given GE at a dose of 50 and 100 mg/kg body weight. The administration of water or GE in rats was provided using a stomach tube only one time 1h 30 min before abdominal surgery. The rats in all groups were sacrificed 5h after the surgery. The entire esophagus was removed immediately and examined for gross mucosal injury. The esophageal tissue was then immediately frozen in liquid nitrogen, serum was extracted from the collected blood, and both were subsequently stored at −80°C with the serum samples until the analysis.

### 2.7. Esophageal Lesion Score

After euthanasia, the esophagus of each rat was cut in a longitudinal direction from the gastroesophageal junction to the pharynx using scissors. The inner mucous was washed away with 0.9% sodium chloride (NaCl) and the remaining tissue was laid out on paper. Then, the dissected esophagus was photographed using an optical digital camera (Sony, Tokyo, Japan) and analyzed using the i-Solution Lite software program.

The gross mucosal damage ratio was calculated as follows: the gross mucosal damage ratio

(3) $$\begin{array}{c} \left(\%\right)=\left[\frac{\text{width of area with esophageal mucosal damage }\left({\text{mm}}^{2}\right)}{\text{width of total area of esophagus }\left({\text{mm}}^{2}\right)}\right]. \end{array}$$

### 2.8. Histological Examination in the Esophagus

For microscopic evaluation, the opened esophagus was cut to isolate the middle segment. This segment was fixed in 10% neutral-buffered formalin and, after embedding in paraffin, cut into 2 *μ*m sections and stained using hematoxylin and eosin (H&E) and periodic acid schiff (PAS). The stained slices were subsequently observed under an optical microscope and analyzed using the i-Solution Lite software program (Innerview Co., Korea).

### 2.9. Measurement of ROS Level in the Serum

The ROS levels were measured by using the method described by Ali et al. [19]. A total of 25 mM DCF-DA was added to the serum sample. After incubation for 30 min, changes in fluorescence were determined at an excitation wavelength of 486 nm and an emission wavelength of 530 nm.

### 2.10. Preparation of Cytosol and Nuclear Fractions

Protein extraction was performed according to the method of Komatsu with minor modifications [20]. Esophageal tissues for cytosol fraction were homogenized with ice-cold lysis buffer A (250 mL) containing 10 mM HEPES (pH 7.8), 10 mM KCl, 2 mM MgCl2, 1 mM DTT, 0.1 mM EDTA, 0.1 mM PMSF, and 1250 *μ*L protease inhibitor mixture solution. The homogenate incubated at 4°C for 20 min. And then 10% NP-40 was added and mixed well. After centrifugation (13,400 ×*g* for 2 min at 4°C) using Eppendorf 5415R (Hamburg, Germany), the supernatant liquid (cytosol fraction) was separated by a new e-tube. The left pellets were washed twice by buffer A and the supernatant was discarded. Next, the pellets were suspended with lysis buffer C (20 mL) containing 50 mM HEPES (pH 7.8), 50 mMKCl, 300 mM NaCl, 1 mM DTT, 0.1 mM EDTA, 0.1 mM PMSF, 1% (v/v) glycerol, and 100 *μ*L protease inhibitor mixture solution suspended and incubated at 4°C for 30 min. After centrifugation (13,400 ×*g* for 10 min at 4°C), the nuclear fraction was prepared to collect the supernatant. Both cytosol and nuclear fractions were kept at −80°C before the analysis.

### 2.11. Immunoblot analyses

To estimate nuclear factor-erythroid 2-related factor 2 (Nrf2), NF-*κ*Bp65, c-Jun, c-Fos, and histone levels, 15 *μ*g of protein from each nuclear fraction was separated using 8–10% sodium dodecyl sulfate polyacrylamide gel electrophoresis (SDS-PAGE). Separated proteins were transferred onto a nitrocellulose membrane, blocked with 5% (w/v) skim milk solution for 1 h, and then incubated with primary antibodies (against Nrf2, NF-*κ*Bp65, and histone) overnight at 4°C. The blots were washed and incubated with anti-rabbit or anti-mouse IgG horseradish peroxidase- (HRP-) conjugated secondary antibody for 1 h. In addition, 10 *μ*g of protein of each cytosolic fraction of HO-1, catalase, GPx-1/2, I*κ*B*α*, p-I*κ*B*α*, COX-2, iNOS, TNF-*α*, IL-1*α*, NOX4, p47^phox^, Rac-1, keap1, p38, p-p38, ERK, p-ERK, JNK, p-JNK, survivin, cytochrome c, caspase-3, Bax, Bcl-2, and *β*-actin was separated using 8–15% SDS-PAGE. The antigen-antibody complex was visualized using ECL Western Blotting Detection Reagents and detected using chemiluminescence with the Sensi-Q 2000 Chemidoc imager (LugenSci Co., Ltd., Gyeonggi-do, Korea). Band intensities were measured using ATTO densitograph software (ATTO Corporation, Tokyo, Japan) and quantified as the ratio to histone or *β*-actin. The protein levels of the groups are expressed relative to those of the normal rats (represented as 1).

### 2.12. Statistical Analysis

The data are expressed as the means ± standard error of the mean (SEM). Significance was assessed using a one-way analysis of variance (ANOVA) followed by Dunnett's multiple comparison test using the statistical package for the social sciences (SPSS) version 22.0 software (SPSS Inc., Chicago, IL, USA). Furthermore, *p*-values < 0.05 were considered significant.

## 3. Results and Discussions

In modern medicine, RE is a multifactorial disorder and esophageal pathology most frequently occurring in gastrointestinal disease and has a significant impact on the quality of life and medical costs [21, 22]. The main pathogenesis of RE is complex and diverse, including esophageal mucosal irritability, decreased esophageal capacity, decreased esophageal sphincter function, and gastric motility disorders [23]. The regurgitation of stomach contents is known to cause impairment, inflammation, ulceration, and necrosis of the normal squamous epithelium of the esophagus [24]. Reflux esophagitis is associated with esophageal stricture and Barrett's esophagus and is likely to develop into esophageal cancer [25]. Drugs such as histamine type II receptor antagonists (H2-RA) or proton pump inhibitors (PPI) have been used to treat reflux esophagitis effectively, but many patients fail to complete mucosal healing and progress to a complex condition or suffer [26].

Recently, antioxidants have been shown to be effective in reflux esophagitis by suppressing symptoms of esophageal mucosa due to excessive free radicals produced in the esophagus [27]. The Gardeniae Fructus has been demonstrated as an effective pharmacological action, including a protective activity against oxidative damage, as well as having cytotoxic and anti-inflammatory effects [28]. Geniposide, the main compound of Gardeniae Fructus, has been reported to have a significant effect on inflammation, ulceration, and diabetes [29]. Herbal remedies are considered to be harmless and nontoxic to humans, suggesting alternatives to existing medicines [30]. The components of the major compound (geniposide) were detected from the Gardeniae Fructus extract. Representative HPLC results are illustrated in Figure 1. The amount of geniposide was as follows: GE: 0.154 g/mL. Geniposide has been shown to reduce inflammatory cytokines in intestinal diseases [31]. In this study, we suggest that geniposide may reduce inflammation in reflux esophagitis.

**Figure 2:**
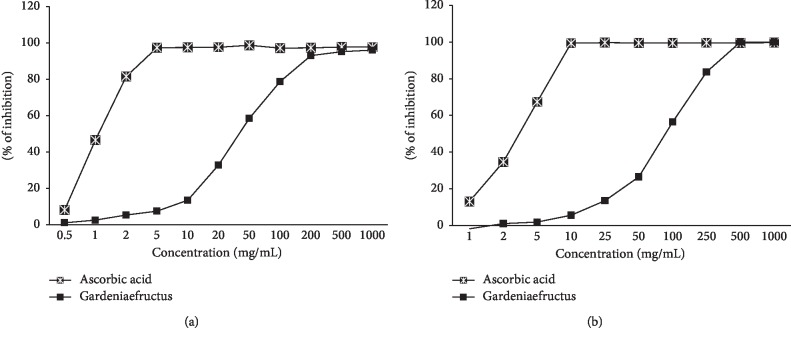
DPPH radical scavenging activity (a) and ABTS radical scavenging activity (b) of GE; each experiment was run in triplicate.

The result of this study was to investigate the antioxidant effect of 50% ethanol extracts of Gardeniae Fructus on the improvement of reflux esophagitis. The DPPH radical scavenging ability and the ABTS radical scavenging test are methods for measuring the antioxidative activity of a sample by an in vitro assay method [32]. In this respect, the DPPH and ABTS radical scavenging IC_50_ values of the GE were measured (41.86 ± 1.35 and 89.57 ± 1.78 *μ*g/mL, resp.) and L-ascorbic acid was used as a standard substance in terms of IC_50_ values (1.15 ± 0.06 and 3.67 ± 0.07 *μ*g/mL, resp.), used as an antioxidant reference molecule. The in vitro antioxidant analysis showed that the GE could prevent oxidative stress induced by RE (Figure 2).

**Figure 3:**
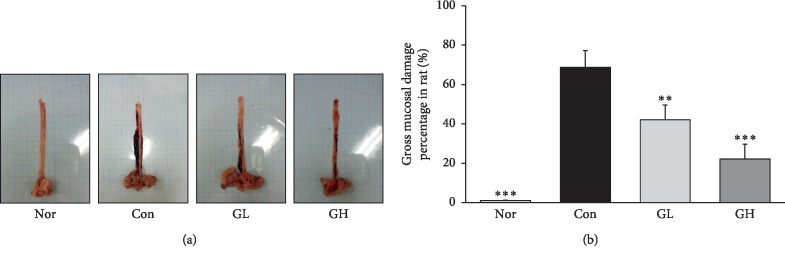
(a) Effects of GE on optical change of esophagus tissues and (b) effects on the gross mucosal injury ratio change of esophagus tissues of reflux esophagitis rats. Nor: normal rats, Con: reflux esophagitis control rats, GL: GE 50 mg/kg treated reflux esophagitis rats, and GH: GE 100 mg/kg treated reflux esophagitis rats. All data are expressed means ± SEM, (*n* = 6) rats per group. Significance: ^*∗∗*^*p* < 0.01,^*∗∗∗*^*p* < 0.001 versus RE control rat values.

Esophageal mucosa is one of the important defense mechanisms through the epithelium (mucous and bicarbonate ions), the epithelial cells (epithelial cells), and the deep epidermis (blood vessel), and the mucosal layer is damaged by the refluxed stomach contents [33]. The esophageal lesion score was significantly increased in the control group compared to the normal group, but, compared with the control group, there was a dose-dependent decrease in the GE treated group (Figure 3).

**Figure 4:**
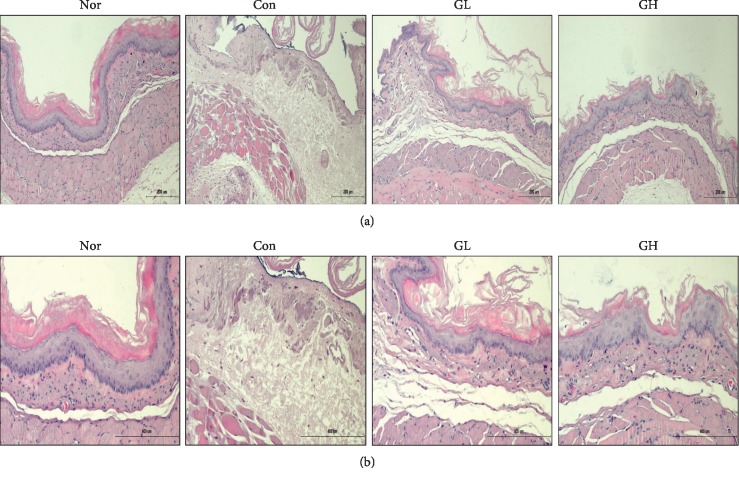
The effect of GE on the histopathological change epididymis esophagus tissues of RE rats. Esophagus tissues were stained with H&E (Original magnification ×200 and ×400). Nor: normal rats, Con: reflux esophagitis control rats, GL: GE 50 mg/kg treated reflux esophagitis rats, and GH: GE 100 mg/kg treated reflux esophagitis rats.

Chronic acid reflux esophagitis is contrasted with the features of hemorrhage, erythema, and multiple erosion in modeled rats and acute reflux esophagitis [34]. In the normal group, lesions were not observed in the esophageal mucosa but the esophageal mucosa was found to be damaged in the reflux esophagus induced tissue. The esophagus showed thickening of the basal layer, inflammatory cell infiltration into the mucosal layer, and exfoliated epithelial cells [2]. Importantly, in the case of the GE group, a concentration-dependent decrease of the squamous layer cell damage and recovery from the inflammation were observed. The degree of damage of esophageal mucosa was dose dependent. Especially, the group treated with 100 mg/kg of Gardeniae Fructus extract showed significant improvement (Figure 4).

**Figure 5:**
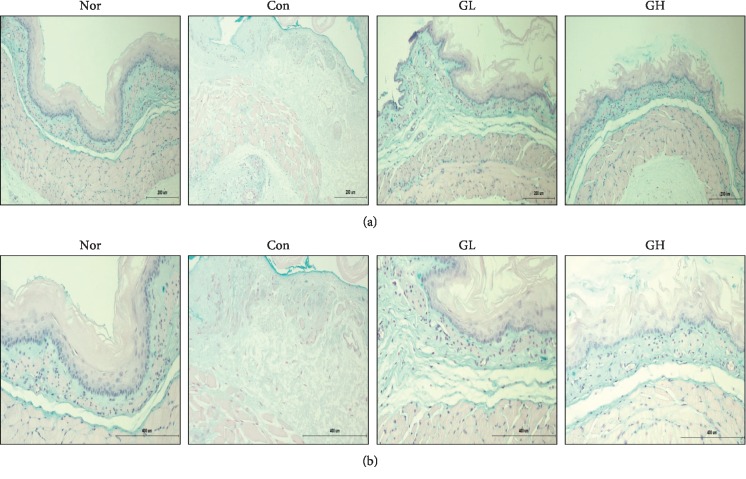
The effect of GE on the histopathological change epididymis esophagus tissues of RE rats. Esophagus tissues were stained with PAS (Original magnification ×200 and ×400). Nor: normal rats, Con: reflux esophagitis control rats, GL: GE 50 mg/kg treated reflux esophagitis rats, and GH: GE 100 mg/kg treated reflux esophagitis rats.

The effects of GE treatment were confirmed by the H&E staining histological characterization in esophagus tissue. In addition, PAS staining showed goblet cells with regular mucosal construction secreting mucus in the GL and GH groups compared to the control group. This observation suggests that GE treatment can be effective against acute reflux esophagitis by promoting mucus secretion (Figure 5).

**Figure 6:**
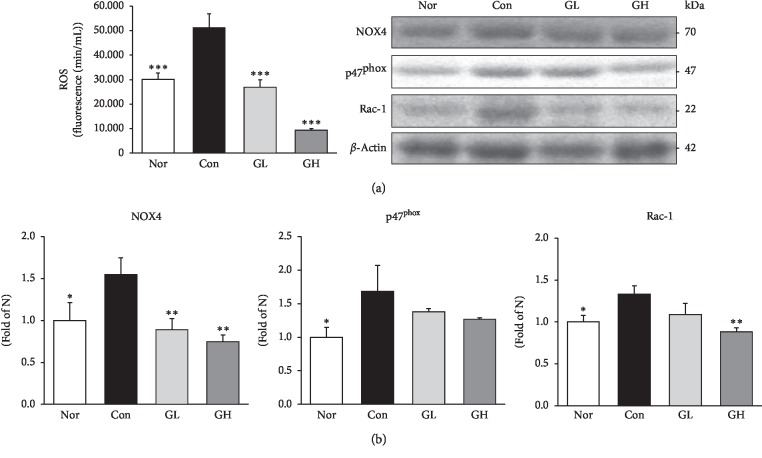
GE decreased serum ROS and NADPH oxidase activity protein expressions. (a) Serum ROS. (b) NOX4, p47^phox^, and Rac-1 protein expressions. Nor: normal rats, Con: reflux esophagitis control rats, GL: GE 50 mg/kg treated reflux esophagitis rats, and GH: GE 100 mg/kg treated reflux esophagitis rats. All data are expressed means ± SEM, (*n* = 6) rats per group. Significance: ^*∗*^*p* < 0.05,^*∗∗*^*p* < 0.01,^*∗∗∗*^*p* < 0.001 versus RE control rat values.

The production of ROS causes cellular damage, and it has been reported to play a role in the pathogenesis of various gastrointestinal diseases [35, 36]. ROS produced by gastric reflux has been shown to cause damage to the esophagus, and these results suggest that the antioxidant activity can prevent tissue damage [37]. In the present study, the elevated levels of serum ROS were significantly decreased lower to the levels of the control group, both GL and GH (*p* < 0.001) (Figure 6(a)).

Nicotinamide adenine dinucleotide phosphate (NADPH) oxidase is an enzyme that catalyzes the formation of ROS [38]. NADPH is a factor associated with tissue damage in inflammatory diseases, with excess production of ROS through NOX4, p47^phox^, and Rac-1 [39]. The protein expressions NOX4, p47^phox^, and Rac-1, the markers of NADPH oxidase activity, in the esophagus were significantly augmented in the RE control group. However, the GL and GH treated groups had significantly downregulated NOX4 and p47^phox^ whereas Rac-1 tended to decrease with the dose-dependence of the GL and GH treatment groups (Figure 6).

**Figure 7:**
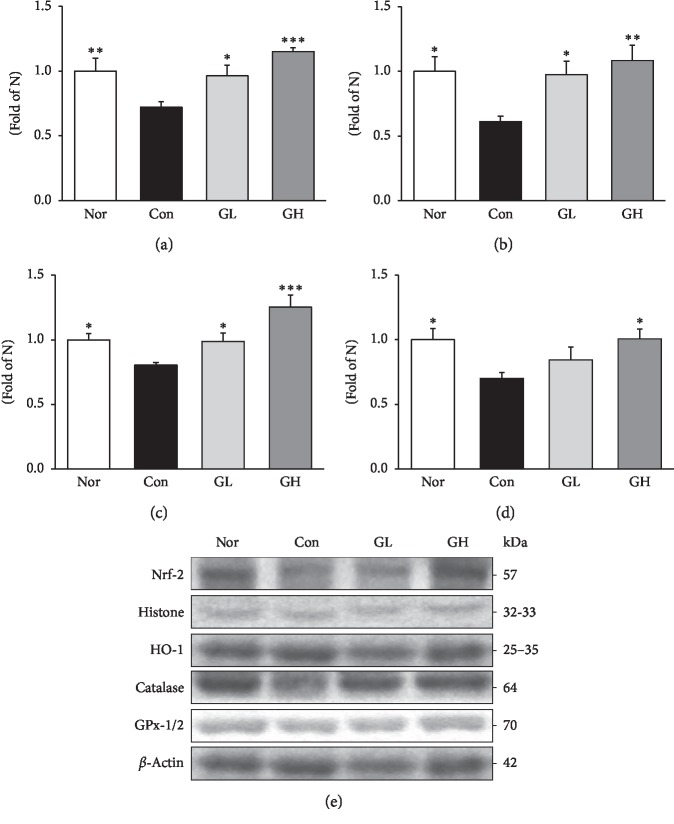
Antioxidant enzyme-related protein expressions in the esophagus. Western blot analysis of (a) Nrf-2, (b) HO-1, (c) catalase, and (d) GPx-1/2 expression. Nor: normal rats, Con: reflux esophagitis control rats, GL: GE 50 mg/kg treated reflux esophagitis rats, and GH: GE 100 mg/kg treated reflux esophagitis rats. (e) All data are expressed means ± SEM, (*n* = 6) rats per group. Significance: ^*∗*^*p* < 0.05,^*∗∗*^*p* < 0.01,^*∗∗∗*^*p* < 0.001 versus RE control rat values.

ROS is generally reported to be neutralized by antioxidant enzymes [40]. In general, Nrf-2 is present in the cytoplasm and oxidative stress induces Nrf-2 translocation into the nucleus. Nrf-2 binds to antioxidant response elements and induces transcription of antioxidant enzymes such as HO-1, catalase, and GPx [41]. The oral administration of GE showed a significant dose-dependent increase in the expression level of Nrf-2 and a significant dose-dependent increase in the activity of HO-1 and catalase. GPx-1/2 activity was also significantly increased in the GH group compared to the control group. The administration of GE suggests that it can upregulate antioxidant factors and effectively remove oxidative stress induced by reflux esophagitis (Figure 7).

**Figure 8:**
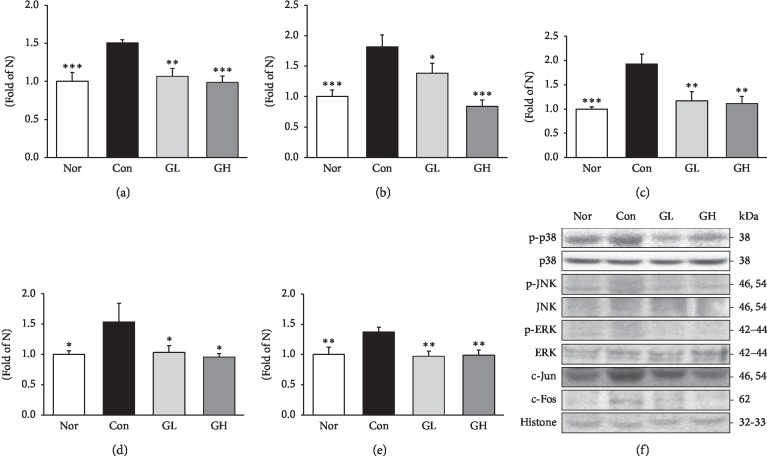
Western blot analysis of (a) c-Jun, (b) c-Fos, (c) p-p38, (d) p-JNK, and (e) p-ERK expressions. Nor: normal rat, Con: reflux esophagitis control rats, GL: GE 50 mg/kg treated reflux esophagitis rats, and GH: GE 100 mg/kg treated reflux esophagitis rats. (f) All data are expressed means ± SEM, (*n* = 6) rats per group. Significance: ^*∗*^*p* < 0.05,^*∗∗*^*p* < 0.01,^*∗∗∗*^*p* < 0.001 versus RE control rat values.

Mitogen-activated protein kinase (MAPK) is the most commonly studied pathway for inflammatory signals. This pathway is activated when extracellular or intracellular stress is present [42]. MAPK (p38, ERK, and JNK) makes a role in making inflammation through the interaction of three pathway factors [43]. JNK activates and phosphorylates c-Jun by oxidative stress [44]. MAPK signaling is associated with nuclear factor NF-*κ*B, one of the transcription factors. In particular, the phosphorylation of p38 and ERK induces activation of NF-*κ*B with many direct and indirect interactions [45, 46]. Both GL and GH treatment groups showed significant results, indicating a role in reducing inflammatory factors (Figure 8).

**Figure 9:**
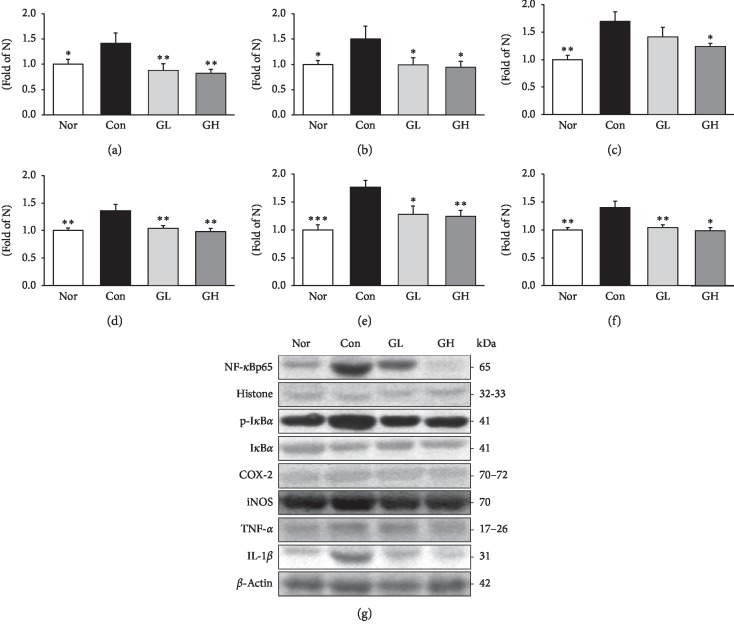
Western blot analysis of (a) NF-*κ*Bp65, (b) p-I*κ*B*α*, (c) COX-2, (d) iNOS, (e) TNF-*α*, and (f) IL-1*β* expressions. Nor: normal rats, Con: reflux esophagitis control rats, GL: GE 50 mg/kg treated reflux esophagitis rats, and GH: GE 100 mg/kg treated reflux esophagitis rats. (g) All data are expressed means ± SEM, (*n* = 6) rats per group. Significance: ^*∗*^*p* < 0.05,^*∗∗*^*p* < 0.01,^*∗∗∗*^*p* < 0.001 versus RE control rat values.

NF-*κ*B regulates immune and inflammatory responses as well as important nuclear transcription factors and regulates several important physiological processes. Due to various stimuli, I*κ*B*α* binds to NF-*κ*B and inhibits NF-*κ*B migration into the nucleus and inhibits its activity [47]. NF-*κ*B participates in controlling the activation of various proinflammatory mediators such as iNOS and COX-2 and cytokines such as IL-1*β* and TNF-*α* [48]. Protein expression of NF-*κ*Bp65 and p-I*κ*B*α* was significantly decreased in the GL and GH treated groups compared to the control group. In the case of COX-2, iNOS, TNF- *α*, and IL-1*β*, the GE treatment group showed a significant decrease in concentration-dependent manner (Figure 9).

**Figure 10:**
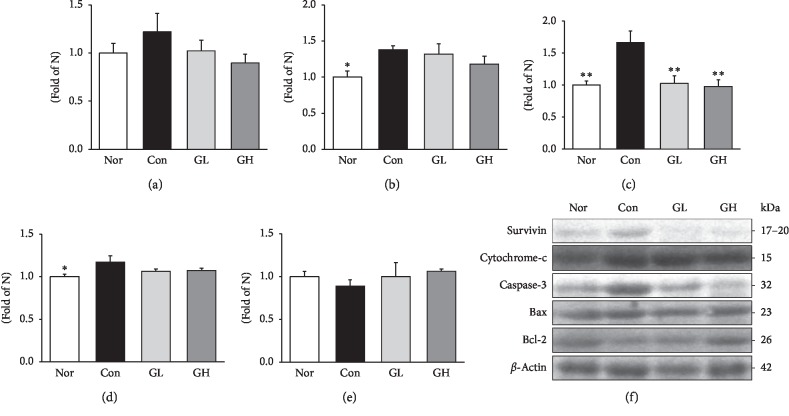
Western blot analysis of (a) survivin, (b) cytochrome c, (c) caspase-3, (d) Bax, and (e) Bcl-2 expressions. Nor: normal rats, Con: reflux esophagitis control rats, GL: GE 50 mg/kg treated reflux esophagitis rats, and GH: GE 100 mg/kg treated reflux esophagitis rats. (f) All data are expressed means ± SEM, (*n* = 6) rats per group. Significance: ^*∗*^*p* < 0.05,^*∗∗*^*p* < 0.01 versus RE control rat values.

The development of inflammation by overexpression of ROS causes damage to the cell and increases apoptosis of the cell [49]. It is known that it is involved in the induction of cell death through the activation of survivin, cytochrome c, Bax, and caspase-3 and the activation of Bcl-2, an antiapoptotic protein [50, 51]. The results showed that the GE treatment group tended to improve more than the control group in the acute model (Figure 10).

## 4. Conclusions

Our results show that the Gardeniae Fructus extract contains a considerable amount of geniposide of the flavonoid family and has an excellent antioxidant activity. Gardenia extract has been shown to be effective for acute reflux esophagitis by reducing oxidative stress in the esophagus and various inflammatory factors and protecting the esophageal mucosa. Therefore, it is suggested that GE could be a potential remedy for the treatment of acute reflux esophagitis.
